# Patient views on asthma diagnosis and how a clinical decision support system could help: A qualitative study

**DOI:** 10.1111/hex.13657

**Published:** 2022-11-12

**Authors:** Anne Canny, Eddie Donaghy, Victoria Murray, Leo Campbell, Carol Stonham, Andrew Bush, Brian McKinstry, Heather Milne, Hilary Pinnock, Luke Daines

**Affiliations:** ^1^ Asthma UK Centre for Applied Research, Usher Institute University of Edinburgh Edinburgh UK; ^2^ NHS Gloucestershire Clinical Commissioning Group Gloucester UK; ^3^ Primary Care Respiratory Society (PCRS) Knowle UK; ^4^ Imperial Centre for Paediatrics and Child Health and National Heart and Lung Institute Imperial College London UK; ^5^ Department of Paediatric Respiratory Medicine Royal Brompton Hospital London UK; ^6^ Centre for Population and Health Sciences, Usher Institute University of Edinburgh Edinburgh UK; ^7^ South East GP Unit NHS Education for Scotland Edinburgh UK

**Keywords:** asthma diagnosis, clinical decision support system (cdss), GPs and asthma nurses, patient perspectives, primary care

## Abstract

**Introduction:**

Making a diagnosis of asthma can be challenging for clinicians and patients. A clinical decision support system (CDSS) for use in primary care including a patient‐facing mode, could change how information is shared between patients and healthcare professionals and improve the diagnostic process.

**Methods:**

Participants diagnosed with asthma within the last 5 years were recruited from general practices across four UK regions. In‐depth interviews were used to explore patient experiences relating to their asthma diagnosis and to understand how a CDSS could be used to improve the diagnostic process for patients. Interviews were audio recorded, transcribed verbatim and analysed using a thematic approach.

**Results:**

Seventeen participants (12 female) undertook interviews, including 14 individuals and 3 parents of children with asthma. Being diagnosed with asthma was generally considered an uncertain process. Participants felt a lack of consultation time and poor communication affected their understanding of asthma and what to expect. Had the nature of asthma and the steps required to make a diagnosis been explained more clearly, patients felt their understanding and engagement in asthma self‐management could have been improved. Participants considered that a CDSS could provide resources to support the diagnostic process, prompt dialogue, aid understanding and support shared decision‐making.

**Conclusion:**

Undergoing an asthma diagnosis was uncertain for patients if their ideas and concerns were not addressed by clinicians and were influenced by a lack of consultation time and limitations in communication. An asthma diagnosis CDSS could provide structure and an interface to prompt dialogue, provide visuals about asthma to aid understanding and encourage patient involvement.

**Patient and Public Contribution:**

Prespecified semistructured interview topic guides (young person and adult versions) were developed by the research team and piloted with members of the Asthma UK Centre for Applied Research Patient and Public Involvement (PPI) group. Findings were regularly discussed within the research group and with PPI colleagues to aid the interpretation of data.

## 1 INTRODUCTION

Asthma is a chronic respiratory disease accounting for at least 6.4 million primary care consultations each year in the United Kingdom.^1^ Although a common condition, making a diagnosis of asthma is not always straightforward for clinicians and estimates from primary care suggest that asthma is often misdiagnosed.^2^, ^3^, ^4^ Overdiagnosis can lead to costly, potentially harmful treatment and may affect job and lifestyle decisions; whilst underdiagnosis risks inadequate treatment and avoidable morbidity and mortality.^5^, ^6^

Asthma is a variable condition with different phenotypes meaning that individuals with asthma can experience and present with a range of symptoms of varying severity.^7^ The aim is to demonstrate objective evidence of variability over time, and investigations such as spirometry and fractional exhaled nitric oxide (FeNO) can increase or decrease the likelihood of a diagnosis of asthma. However, in primary care, timely access to tests is not guaranteed and false positive and false negative results are common.^5^ Consequently, it may take months before a clinician feels able to confirm (or refute) a diagnosis of asthma.^8^ The potentially long timescale can lead to frustration and uncertainty amongst patients^9^ and needs to be handled confidently and accurately by clinicians.^10^

In addition to increasing the availability and use of objective tests,^11^ clinical decision support systems (CDSS) could provide a solution for improving the accuracy of an asthma diagnosis and may help improve the patient experience. CDSS are usually designed to aid the decision‐making of clinicians^12^ but may be utilized to facilitate shared decision‐making with patients. For instance, CDSS can be used to collect, calculate and present information about the likelihood of a diagnosis or treatment benefit. For example, AsthmaTuner, a self‐management system which collects lung function and symptom data via Bluetooth spirometer and patient app, respectively, provides automated treatment recommendations for patients and an interface for health professionals.^13^ For diagnosis, a CDSS could be used to calculate the likelihood of a particular condition, and present the probability and options for confirming a diagnosis. In a Norwegian study, a web‐based CDSS designed to aid the diagnosis and classification of chronic obstructive pulmonary disease (COPD) in primary care was found to reduce misdiagnosis and increase the number of patients receiving smoking cessation advice but did not improve the prescription of pharmacological treatment.^14^

Having derived and validated a clinical prediction model for asthma diagnosis,^15^, ^16^ we plan to implement the model in primary care as a CDSS. Being aware that a previous systematic review found that CDSS for asthma were infrequently utilized,^17^ we wanted to understand patient views on asthma diagnosis and how a CDSS could help to maximize the potential value of a future CDSS for patients. Therefore, to inform the development of the CDSS, this study aimed to explore patient experiences regarding asthma diagnosis and to understand how a CDSS could also be used to improve the diagnostic process for patients.

## 2 METHODS

To inform the development of an intervention (the CDSS), the study design was guided by the Medical Research Council (MRC) framework for developing and evaluating complex interventions.^18^ To understand which design features would be most important for an asthma diagnosis CDSS, the experiences and views of patients (and parents of children) who had undergone an asthma diagnosis were sought using qualitative methods. We undertook interviews with young people, adults and the parents of children, who had a recent diagnosis of asthma (ideally within the past 5 years). Interviews took place between 1 October 2020 and 31 January 2021. All participants provided informed consent before interviews were conducted.

### 2.1 Recruitment and sampling

Participants were recruited from general practices across four regions within the United Kingdom (Greater Glasgow and Clyde, Lothian, West Midlands, Yorkshire and Humber). Participating practices identified adults and young people (≥12 years) and parents of children (≥5 years) who had ‘active asthma’ and a diagnosis of asthma coded in the electronic health records within the last 5 years. ‘Active asthma’ was defined as a coded diagnosis of asthma and having had a prescription for any asthma treatment within the previous year.^19^ Children below 5 years of age were excluded as viral‐associated wheeze is common in this age group and can complicate asthma diagnosis. Based on the age of legal consent, we offered young people aged ≥12 years the chance to take part in an interview themselves. A clinician from each practice screened the list of selected patients for eligibility and excluded individuals who had COPD, were unable to give informed consent (e.g., due to cognitive impairment) or for social/clinical reasons (e.g., significant co‐morbidity, recently bereaved or on a palliative care register).

Potential participants were mailed an information sheet and an expression of interest form which included questions about age, gender, age at diagnosis, how asthma was confirmed (i.e., symptoms, examination, tests) and the confidence they had in their asthma diagnosis (agree, not sure, do not agree with diagnosis). Responses to these questions were used to purposively sample individuals to represent a range of participants in terms of age, gender, length of time since asthma diagnosis, confidence in the diagnosis and who made the diagnosis.

### 2.2 Data collection

Semistructured in‐depth interviews were conducted by telephone (to comply with social distancing during the COVID‐19 pandemic). Interviews were conducted by a male (E. D.) or female (V. M.) postdoctoral researchers, both of whom have experience in health services research. Interviews lasted between 30 and 45 min and were audio recorded, transcribed verbatim and redacted of identifiable information. No repeat interviews were necessary. Transcripts were not returned to participants for comment.

### 2.3 Topic guides

Prespecified semistructured interview topic guides (young person and adult versions) were developed by the research team (see Supporting Information). To consider the validity and reliability of the topic guides, we conducted pilot interviews with members of the Asthma UK Centre for Applied Research Patient and Public Involvement (PPI) group. Topic guides mapped to the study objectives and were designed to allow a focused yet flexible approach^20^ that facilitated exploration of experiences of asthma diagnosis; perceptions and expectations of patient involvement in the diagnostic process and how a computer system/CDSS could have helped or hindered their experiences.

### 2.4 Data analysis

We used a thematic approach to data analysis.^21^ Using Nvivo 10 (QSR International), transcripts were read and manually coded using overarching themes. In an attempt to maximize reliability, after the initial transcripts had been coded, three researchers (V. M., L. D., H. P.) conducted a thematic analysis with selected transcripts during this iterative process. Emerging themes were discussed before deciding on an initial coding framework. Transcripts were coded on an ongoing basis concurrently with interviews and revisited as the study progressed so that new themes could be included, and the coding framework refined. The final coding framework was thus a combination of themes proposed in advance together with other themes generated during the analysis,^21^ which represents both a deductive and inductive approach to qualitative analysis.^22^ The consolidated criteria for reporting qualitative research was used to guide reporting (see Supporting Information).^23^

### 2.5 Interpretation

We took a critical‐realist perspective when interpreting the data,^24^ which helped when considering the experiences, motivations and meanings of participants' lived realities.^21^ To aid interpretation, findings were regularly discussed within the research group and with PPI colleagues. The concept of medical dominance emerged as relevant, and we used this to guide interpretation.^25^, ^26^ Medical dominance is based on the view that in relation to health and illness, medical professionals hold power and control which shape and influence health professional/patient interactions and experiences.^27^, ^28^, ^29^

## 3 RESULTS

We received 53 expressions of interest within the study period and using purposive sampling, 27 individuals were invited to take part. 17 participants contributed to interviews, including 14 individuals with asthma and 3 parents of children with asthma (Table 1). All participants had been diagnosed with asthma before the COVID‐19 pandemic. Of the 26 individuals not invited, 23 had been diagnosed several years before and 3 children had been diagnosed before 5 years of age.

**Table 1:** Patient demographics

Characteristics	Participant (*n*)
Sex	
Female	12
Male	5
Age (years)	
<16	3
16–30	2
31–40	3
41–50	5
51–60	2
61–70	2
Years since diagnosis (years)	
<2	3
2–4	7
4–6	1
6–8	1
8–10	3
>10	2
Diagnosis made by	
Asthma nurse	3
General practitioner	10
Hospital staff	4
Confidence about their asthma diagnosis?	
Yes	12
Not sure	5
Parents of children interviewed	3
Location (type, practice size)	
Site 1 (urban, 10,361)	5
Site 2 (urban, 6576)	4
Site 3 (urban, 5465)	1
Site 4 (urban, 10,123)	4
Site 5 (semiurban, 12,578)	2
Site 6 (urban, 3851)	1

### 3.1 Overview of themes

Analysis of data sought to answer two key research questions: patient experiences during an asthma diagnosis and patient views and experiences of a CDSS. Four subthemes were identified regarding patient experiences during the diagnostic process; knowledge and understanding of asthma, communication, receiving and retaining information and self‐management. An additional four themes emerged in relation to patient experiences and views of a CDSS; patient experiences of screen sharing, online health information use, patient views on an asthma CDSS and barriers and facilitators to a CDSS being used. Topics are reported in this order.

### 3.2 Key theme 1: Diagnosis: The patient experience

#### 3.2.1 Knowledge and understanding of asthma

Several participants recalled being uncertain about what asthma was or how it might present. For instance, participant adult/1 (female, age 41–50, site 1), recalled that she ‘didn't know that coughing was a sign of asthma’, despite having a sister and a best friend who had been diagnosed with the condition as teenagers. Other participants believed asthma always started in childhood:

It was a bit weird 'cause I'd never had it before and [obviously it] was … like, I thought it was quite late. I thought it was one of those things you just had as a kid and then, like, you had it from the beginning and that was that. (P/young person/1, female, 16–30, site 5)

Participants often had their own ideas about the cause of symptoms, and without prior knowledge or experience of asthma, some individuals worried about what they viewed as the worst‐case scenario such as cancer:

Somehow you associate it (asthma) with really sick people. I don't know. I didn't sort of think of it as a kind of a manageable issue. Sort of, these people who had maybe asbestos poisoning to their lungs or something like that. A very dramatic thing. (P/adult/6, male, 41–50, site 1)

In a similar vein, some participants, held a lack of familiarity with asthma symptoms leading them to assume their symptoms were a consequence of lifestyle choices or personal stresses so achieving a diagnosis was a relief:

We bought a house which we then discovered had a lot of hidden mould issues and I think that's been a contributor to all of this [….] The asthma diagnosis really helped. (P/adult/6, male, 41–50, site 1)

For some participants, an asthma diagnosis came as a surprise and was made co‐incidentally during an appointment for another problem:

I was actually diagnosed accidentally, but I was glad I was diagnosed at the time. I was actually meant to go about my toe because I was arguing with my son when he was about five years old and I got my toe jammed under the door and it was bruised. So anyway, I went to the doctor about it and he noticed that I was a bit wheezy so he decided that he would do a test. And he turned round and said, yeah, you're asthmatic. (P/adult/4, female, 41–50, site 2)

#### 3.2.2 Communication

The importance of communication during the assessment for an asthma diagnosis was a common theme arising from interviews. Some participants were surprised that being diagnosed with asthma had taken a long time, and another participant remained unsure if they had asthma:

Cause all along they're like, oh there's no official test so this might not be, so you'll just need to try this and try that and see if it works or not. So, it's quite a … like, unsure and quite a long process sometimes. (P/young person/1, female, 16–30, site 5)

There was never any concrete diagnosis, so I don't know whether I have a pre‐existing condition now or not. (P/adult/9, male, 41–50, site 4)

However, many patients were satisfied with the step‐by‐step processes they experienced and the principles of parsimony by problem‐solving through the simplest means available to enable an accurate diagnosis:

‘It took a while. It was sort of an ongoing thing over … well, I'd had sort of recurring colds and kind of persistent things like that, with kind of wheezing and coughing and so on. And […] my GP, I think was quite methodical about this so there were various tests and eliminations and so on. So, it had come over a couple of years’. Yeah, but I think it's the right way to do it. I was very happy with it. (P/adult/6, male, 41–50, site 1)

A lack of communication about the variable nature of asthma during the early stages of the diagnostic process left some respondents unsure if they had asthma or not. P/young person/2, reported feeling ‘a wee bit shocked’ when diagnosed with asthma because she considered herself to be ‘the fittest I'd ever been’. Participant adult/8 (male, 61–70, site 3) was told he had asthma but said ‘it wasn't explained how my lungs work or anything’. Participant young person/2, felt disappointed with the support she had received, explaining she had been ‘just told and then left with that information’:

You sometimes feel that people are just giving you a decision but not explaining it in enough detail. [….] Even like when my mum's been there with me, it's just been, kind of […] like none of us have fully understood how I have asthma. (P/young person/2, female, 16–30, site 2)

A perceived lack of time and the use of complex language were reasons participants gave for communication being poor. Participants generally understood why time may be an issue. For instance, P/adult/6 (male, 41–50, site 1), who had been diagnosed for 1 year, felt that ‘the system is congested’. Similarly, P/adult/2 (adult, female, 21–30, site 1), talked about General Practitioners (GP) as being ‘obviously very busy’ and her GP did not have time to explain the diagnostic process or use language that the patient understood. Consequently, she preferred to see the asthma nurse who provided ‘more of an understanding about what's going to happen’:

I think the thing about the nurses are … especially the asthma nurses, they, kind of, already […] I mean, they only see you once a year but they know you a bit better so they can, kind of, explain things in a bit more user‐friendly way, I guess. (P/adult/2, female, 21–30, site 1)

Parents of children undergoing the diagnostic process felt communication was particularly problematic, and reported feeling frustrated and helpless during a lengthy and often inconclusive testing process:

You know, I was really annoyed because, you know, like every second week she was ill and [had a] high temperature. And like even, you know, we went to emergency hospital appointment, and nobody could say nothing. And I thought because she was … she has a twin sister, and they were born two weeks before due. Yeah, and […], another problem, like, because she is the second twin, she's the youngest one. And when she had cough and the doctor said, because (twin 1) has asthma, (twin 2) probably has asthma as well. And for me it's like, she never had a test. (P/parent of child/2, female, age 9, site 2)

#### 3.2.3 Receiving and retaining information

Whilst one participant (P/adult/4, female, 41–50, site 2) reported her diagnosis was explained to her satisfaction and she remembered everything she was told, most respondents reported leaving their appointment(s) with little information or not being able to recall the information later. For some respondents, much of the asthma information received was new to them and felt overwhelming. One individual felt relief at finally having a label for their condition. Others suggested they had appeased their GP by pretending to follow the conversation:

It takes me sometimes a while to cotton on to things. And I may say, ‘yes, I understand’ at first. I suppose I'm fairly typical of most people in that way. I say ‘yes, I understand’ but I don't think I've got a full grasp of it, you know? (P/adult/3, male, 61–70, site 1)

The provision of paper handouts to support asthma information during consultations was useful for some, met with indifference by others, and received poorly by one or two participants. Handouts were unlikely to be kept and the information on them was not well retained:

I probably got a leaflet or something like that, that had two or three pages in it, then, and then, well, a leaflet, you put it down and then it disappears. (P/adult/7, female, 51–60, site 1)

I'm a digital person. I hate bits of paper, 'cause I lose bits of paper. (P/adult/10, male, 61–70, site 4)

#### 3.2.4 Self‐management

In keeping with the perceived lack of information provided at the time of diagnosis, some individuals felt underconfident in managing their asthma after they had been diagnosed, for example, taking their inhaler correctly:

The only problem, if it could be said to be a problem, was I didn't know how to use the inhalers correctly. I don't believe, I don't remember being told how to use an inhaler. (P/adult/3, male, 61–70, site 1)

Although some participants talked about their personal asthma action plan (a key component of asthma self‐management),^5^ several respondents said they had not been provided with one, and others did not know what they were:

I know everybody talks about their asthma plan, but mine is not like … I've not got any asthma plan written down, but I mean […] I know myself and I've got an oximeter in the house as well now that I will test on these various things. (P/adult/13, female, 41–50 site 4)

### 3.3 Key theme 2: CDSS: Patient experience and views

Participants spoke about a range of topics relating to how a computer, the internet or a CDSS could be used to enhance a consultation for a possible asthma diagnosis. Four subthemes were identified: patient experiences of screen sharing, online information use, patient views of an asthma CDSS and barriers and facilitators to CDSS use.

#### 3.3.1 Patient experiences of screen sharing

Respondents were asked about their experiences of using the screen alongside their clinician during appointments.^30^ One or two participants talked about screen sharing with their asthma nurse, but most could not recall being invited to look at the computer screen during a GP consultation. Few participants realized that they could be invited to look at the screen, or even understood why they might want to see it:

GPs certainly not, I don't think they ever share screens. The asthma nurse … I think they have like, they've shown us, but they are just graphs, not really to do with asthma necessarily. They are to do with like height and weight and where you should be and then your peak flow, that stuff. (P/parent of child/1, male, age 14, site 4)

Well, I don't really think like that is a nice thing to do … Aye, I'm just thinking that (screen) was a bit private, you know, would that not be a bit private to them? (P/adult/12, male, age 51–60, site 6)

That said, some participants had experience screen sharing during clinical consultations:

Certainly, in the hospital in most sessions. I'm quite curious as an individual anyway, and dangerous because I have a little bit of knowledge, so I've been looking at the numbers they were copying down. I think in the consultant conversation he was definitely pivoting the monitor so we could look at it. I can't remember what was on it, but I do remember that seating arrangement to both look at it. (P/adult/9, male, 41–50, site 4)

Some respondents felt that a CDSS which allowed them to see how the clinician worked through their diagnosis, might have helped them to understand more about the variability of asthma and other aspects to help understand the condition.

So, yes anything that provides better, broader information from a multitude of directions, so not just ‘Here is a piece of writing for you’. Like you are seeing with visuals, you know, I think is only going to make it better. (P/parent of child/1, male, age 14, site 4)

#### 3.3.2 Online health information use

When asked about accessing health information online pre‐diagnosis, a lack of trust in the quality of information online alongside patient perceptions of GP dislike of the practice, meaning that most respondents avoided using the internet to try and self‐diagnose.

I think I'm of the generation that what the doctor tells me I believe him. I tend not to look up illnesses myself. (P/adult/11, female, 61–70, site 4)

I don't go online so much […] because I work for a health organisation. And I know that doctors get annoyed with, sort of, patients looking up symptoms online before actually going to see them; and then thinking they've got something when they've not actually got it. So, that's maybe one of the reasons I don't tend to sort of go online to look out for health problems and things like that. (P/adult/1, female, 41–50, site 1)

However, participants noted that they accessed information online post‐diagnosis to expand their knowledge or define their condition better:

I looked it up, which I never normally do, asthma symptoms. And it's because I was still coughing and I'm … the thing I says to my brother and sister, I'm not convinced I've got asthma. I think it's a chest infection […] So … what I read on the Internet, sadly […] confirmed what I was feeling [was asthma]. (P/adult/8, male, 61–70, site 3)

There was a perception that negative clinical attitudes existed towards patients exploring online information before a GP diagnostic appointment (P/adult/1, female, 41–50, site 1). Conversely, some held the view that ‘Dr Google’ was useful particularly in terms of searching for groups to exchange views and experiences of asthma.

Everything seems to be online, and everybody seems to have an opinion and so easily accessible […] information that you need, and you know, you've got your asthma, you know, groups online. (P/adult/13, female, 51–60, site 4)

#### 3.3.3 Patient views on an asthma CDSS

The most popular output for the CDSS was the ability to provide the probability of an asthma (ideally visually) during diagnostic consultations. Moreover, respondents agreed that being able to see the factors which could lead towards an asthma diagnosis would be useful alongside further information to improve treatment management:

And he could say, I don't know, let's say there's various fields on your screen, if five out of these ten fields are ticked, the chances are, that you've got asthma or whatever disease and as you can see you've got seven of them ticked; you know, something like that. A visual representation. (P/adult/10, male, 61–70, site 4)

In keeping with the lack of confidence that individuals had about their understanding of asthma, participants suggested that incorporating an educational section within the CDSS which could be used during the consultation to show a visual representation of how asthma affects the lungs would be of interest and could assist communication and their understanding:

I actually think that kind of thing would be really helpful for children and young people…. because it's very abstract, and especially if it's just something that you think is just how your body is, you never question it, you never really think about it in terms of the actual physiological processes that are happening, you are just like, ‘Oh I've got asthma, right’. (P/parent of child/1, male, age 14, site 4)

If there's a simulation or something like that, ‘Here's how it looks when it's really bad’ and ‘Here's how, what’, ‘Here's how an inhaler, what it does to your lungs’, ‘Here's what specific medication does’ and stuff like, yeah, I think that would be very interesting […] just looking at it on a piece of paper, is not the best. I think seeing some kind of simulation would be much more helpful. (P/adult/7, female, 51–60, site 1)

Respondents also noted that it would be beneficial to understand where they fit into an overall picture of asthma severity, with P/adult/9 (male, 41–50, site 4), two years since diagnosis asking, ‘What's normal and where am I versus normal?’ Some participants felt that understanding the significance of their diagnosis could have helped them take the diagnosis more seriously from the beginning. Participants also suggested that a website associated with the CDSS which could be used after the consultation would be more beneficial to them in the long run than the traditional handouts:

I think it's a good idea. I think it would help quite a lot 'cause the big problem I had was that I wasn't using my inhaler correctly and then I wasn't seeing an improvement on … kind of, on my, like, lung capacity essentially. So, I think if I'd, kind of, had that understanding earlier on then I would have been more dedicated to using my inhaler the way that I'm meant to. (P/adult/2, female, 21–30, site 1)

There were others who were sceptical, believing their diagnosis would not have been speedier or different with the aid of a CDSS.

I mean, I have to say that on these indicators alone, my family history was ‘no none’. At that point I didn't really have any allergies, they have come on since. Also, my coughing had, ironically, stopped by the time … after the first episode my coughing had stopped because of the operation. Also, I didn't have a wheeze. (P/adult/13, female, 51–60, site 4)

#### 3.3.4 Barriers and facilitators to a CDSS being used

Respondents expressed interest in the potential role of the CDSS and could see areas where the CDSS might improve the diagnostic experience. However, respondents also highlighted that whether the potential was realized depended on how the CDSS was used:

So, I think this system would be good but if it's just the system and then a very overworked GP that doesn't make eye contact, it's not really going to work. It would be, kind of … you know, you'd have to have the right person who was interacting in … on it with you. (P/adult/2, female, 21–30, site 1)

To this end, most respondents viewed the CDSS as an avenue through which communication between patients and clinicians could be facilitated:

Between yourself and the health professionals, this might be a little bit of a focal point for the conversation. So, I think that's likely to work well. (P/adult/6, male, 41–50, site 1)

The ability to aid understanding between clinicians and patients was viewed as the most important aspect of the CDSS, especially for those respondents who found the initial diagnosis ‘daunting’ (P/adult/7, female, 51–60, site 1). Moreover, screen‐sharing was viewed as an opportunity to be ‘treated like an intelligent adult’ (P/adult/6, adult male, 41–50, site 1). Using the CDSS could provide a framework for clinicians and patients to use together to provide a better‐shared understanding of potential routes to diagnosis.

## 4 DISCUSSION

Being diagnosed with asthma could feel like an uncertain process for participants in this study, who felt that limited consultation time or poor communication made it difficult to understand how and why the diagnosis had been made. Some participants felt they retained information about asthma diagnosis poorly and considered online or digital resources more useful than paper handouts. Participants felt possible advantages of a CDSS for asthma diagnosis may be prompting dialogue, improving understanding and encouraging a shared diagnostic process between patients and clinicians.

### 4.1 Interpretation

The hallmark of asthma is variability. Symptoms vary over time and in severity and making a diagnosis of asthma can require time or repeated investigations to build up the information required.^5^, ^8^, ^9^ For patients, preconceived concepts about what asthma is, and who is at risk of developing the condition influence the credibility of an asthma diagnosis. For clinicians, weighing up the probability of an asthma diagnosis, differentiating between asthma and other conditions and excluding red flags can all influence how a consultation is conducted.^8^, ^10^ Thus, the perceptions of both clinician and patient can shape a consultation and a mismatch in these perceptions may lead to dissatisfaction.^30^ Involvement of patients in consultations to allow shared decision‐making is widely accepted in medical practice and may lead to better asthma control, quality of life, adherence to medication and patient satisfaction.^31^ Yet for shared decision‐making to occur, clinicians need to have time and resources to provide information on the pros and cons of a particular course and patients need to feel able to understand and question the medical explanations while contributing what is important for them.^32^, ^33^, ^34^

Amongst the diagnostic experiences recalled in this study, there were instances where participants felt their diagnosis was a *fait accompli* or did not feel empowered to ask questions or engage in meaningful interactions, believing the professional opinion was final. Additionally, and often because of perceived time constraints during GP appointments, patients were reluctant to ask for clarification or explore their diagnosis further, even though they were often dissatisfied with the information provided. One patient deliberately refrained from seeking further asthma information online, believing that doing so would be annoying for their GP. These examples may indicate a mismatch in the perceptions of health between patients and clinicians during the consultation. One influencing factor may be patient/clinician power imbalance, whereby the health professional was perceived to hold power within the consultation.^26^, ^27^, ^28^ A perceived superiority of clinicians in the eyes of patients can impact their willingness to share their opinions,^34^ and engage in consultation because they trusted that the clinician knew best.^35^ On the other hand, some patients may prefer a more direct consulting style and not be actively involved in decision‐making.^36^

Consequently, strategies/interventions to support a more egalitarian partnership between patient and professional may encourage more supportive patient care, an increased understanding of individual illness and facilitate patient empowerment.^28^, ^29^ In this study, there were a few examples of reduced medical dominance; participants described screen sharing and the layout of seating in the consultation room. In keeping with a prior study^30^; screen sharing did contribute to patients feeling involved in the consultation, yet few participants had direct experience of it occurring. Whilst the theory of medical dominance extends beyond doctors to allied health professionals and nurses,^28^, ^29^ some participants in this study preferred to see their asthma nurse (compared to a GP) because they found the consultation more understandable. Other factors such as the length of the appointment may also have influenced this view.

To promote shared decision‐making, Agoritsas et al.^33^ suggested clinicians need ‘skills and tools’ while patients require ‘information and support’. CDSS has traditionally been seen as technology to support clinicians, but can also promote patient‐focussed practice^37^ through the involvement of patients in decision‐making about their health and well‐being.^38^, ^39^ In this study, participants liked the idea of visualizing the probability of asthma, seeing simulations of lung physiology and being able to see how the clinician worked through their diagnosis. However, some participants had reservations, explaining there would be no point in a CDSS if the clinician did not have time to engage. In keeping with this view, some have argued that the barriers to using CDSS set them up for failure.^40^, ^41^, ^42^ For instance, with a lack of guidance on how decision support systems could be used, clinicians were more likely to rely on their training and experience than on new technologies.^43^ Additionally, a lack of appointment time could result in reduced patient involvement, as the clinician focuses on the CDSS rather than inviting the patient to become part of the decision‐making.^40^

### 4.2 Implications for research and practice

The importance of diagnostic tests was noted by participants in this sample, and the lack of access to tests is a source of frustration and uncertainty for patients and health professionals alike.^10^ In Germany and Sweden, spirometry can be achieved at the time of presentation, or within 2 weeks, respectively.^10^ Yet in other health systems, including the United Kingdom, the time between the first presentation and achieving spirometry or FeNO can be months. Therefore, in the United Kingdom, one implication of this work is to improve capacity and timely access to diagnostic tests for asthma.^44^, ^45^ Digital solutions, such as the AsthmaTuner self‐management system, could transform the diagnosis and management of asthma.^13^, ^46^ The use of connected technologies such as wearable sensors, Bluetooth spirometers and digital peak expiratory flow devices could increase access to diagnostic information and allow measurements to be performed when a patient is symptomatic.^13^, ^46^ CDSS which collates data from such devices and supports interpretation could lead to improvements in the diagnostic accuracy of asthma though further high‐quality studies are needed.

In situations where testing remains difficult to achieve, where the outcome of tests makes the diagnostic process protracted (i.e., false negatives), or variable symptoms occur over an extended timescale, considering how best to achieve shared *diagnosis‐making* through explanation of the current situation and deciding on the most appropriate next steps may help patients remain involved. The role that an asthma diagnosis CDSS may have in engaging patients through the diagnostic process is planned to be evaluated during a feasibility pilot study.

### 4.3 Strengths and limitations

The study was designed and piloted using input from a multidisciplinary advisory group and PPI members to develop topic guides and trial interviews which ensured that the topics covered were important to those with asthma and the study team. We sought views from a wide range of individuals who had been recently diagnosed with asthma from different areas across England and Scotland. Despite invitations being sent to those who had a diagnosis of asthma coded in the electronic health records within the last 5 years, we received many expressions of interest from individuals who had been diagnosed over 5 years before, and from individuals over 50 years of age.

Recruitment was severely hampered by the COVID‐19 pandemic for the following reasons: non‐COVID studies (such as ours) were paused to prioritize urgent research which meant the planned study period was restricted; GP practices faced high workload and reduced staffing which made it more difficult to recruit practices; it took longer for clinical research network staff to gain access to GP practices and send out invitations to patients. Despite these challenges, we managed to recruit parents of children with asthma, young people and older adults, with a range of diagnostic experiences. Having interviewed 14 participants, we considered that with respect to adults over 30 years, no new information was being collected and no new codes were developed.^47^ In line with our purposive sampling approach, we chose to complete further interviews to enhance the diversity of the sample, specifically parents of children and young people. Before the study period closed, we were able to include more parents of children and one participant in the 16–30 age group.^47^ Overall, we felt that data saturation had been achieved because the themes had been fully described with no new information being obtained in the later interviews. However, we acknowledge that had we been able to recruit more males, participants aged 16–30 years and individuals from rural GP practices we may have heard about different experiences.

This study sought views on a proposed CDSS being developed by the research team. Regarding reflexivity, we acknowledge the desire to create a successful CDSS may have influenced data collection and interpretation by being eager to pick up on positive aspects during interviews and identifying favourable opinions when analysing the data. We attempted to minimize the influence of any one individual by having two researchers conduct interviews and several team members (including a steering group) contribute to the interpretation of results.

## 5 CONCLUSIONS

The process of diagnosing asthma was uncertain for patients if their ideas and concerns were not addressed by clinicians and were often related to a perceived lack of consultation time and limitations in communication. A CDSS designed with patients' needs in mind could encourage a more shared diagnostic process between patients and clinicians, and improved communication relating to the nature of the condition and its management, including the patient's role in self‐management.

## AUTHOR CONTRIBUTIONS

Luke Daines and Hilary Pinnock conceived the idea and achieved funding. Victoria Murray and Eddie Donaghy conducted patient interviews. Anne Canny, Eddie Donaghy, Luke Daines, Hilary Pinnock and Victoria Murray wrote the first draft and Leo Campbell, Carol Stonham, Andrew Bush, Brian McKinstry and Heather Milne were involved in the interpretation of data, revising it critically. All authors gave final approval for the article to be published.

## CONFLICT OF INTEREST

The authors declare no conflict of interest.

## ETHICS STATEMENT

The study received ethical approval from London Stanmore Research Ethics Committee (ref: 19/LO/1722).

## Supporting information

Supplementary information.

## Data Availability

The data that support the findings of this study are available on request from the corresponding author. The data are not publicly available due to privacy or ethical restrictions.
