# Supplementary material for: Patient views on asthma diagnosis and how a clinical decision support system could help: A qualitative study
Source: Health Expect. 2022 Nov 12;26(1):307–17. doi: 10.1111/hex.13657 (PMC9854294; doi:10.1111/hex.13657)
Supplement: Supplementary file 1 — Supplementary information. [file HEX-26--s001.docx]

**Patient views on asthma diagnosis and how a clinical decision support system could help: a qualitative study**

# Supplementary Material

# Contents

[Contents 1](#_Toc114768000)

[Asthma CDSS Qualitative Study: Young Person Topic Guide 2](#_Toc114768001)

[Asthma CDSS Qualitative Study: Patient Topic Guide 2](#_Toc114768002)

[Supplementary Table: COREQ (COnsolidated criteria for REporting Qualitative research) Checklist 4](#_Toc114768003)

## Asthma CDSS Qualitative Study: Young Person Topic Guide

**Experiences of diagnosis**

1. Can you describe how you found out that you had asthma?

- Who told you that you had asthma?

1. Did you think that you might have had asthma before you doctor told you?

- Had you or your parents googled symptoms?
- What did you know/think about asthma before the diagnosis?
- Do you know anyone with asthma?
- Did you think it could potentially be something else?
- **IF YES** Do you still think that it could be something else?
- Did you speak to your GP about the possibility of it being something else?

**Tests**

1. During an appointment about your asthma have you ever looked at the computer alongside your GP?

- **IF YES** can you describe the situation in which you were able to view it?
- Who suggested that you look at the computer together?
- What were you able to see?
- Did it help you to understand more about what your GP was telling you? Why/why not?
- **IF NO** why have you never looked at it? Would you ever ask to look at the computer?

1. Can you think of a conversation with your GP in which you might have felt this was useful to look at the computer?
2. Did you feel included when the doctor was trying to decide if you had asthma? Why?

- What did your GP do/say that made you feel included?
- What did your GP do/say that made you feel excluded?

1. What do you think GPs could do differently to help make young people feel included in diagnosis in future?

**Uncertainty**

1. Would seeing the computer alongside your GP make you feel more/less confident in your diagnosis?
2. Why/why not?
3. What would it be useful for the computer system to be able to show patients?

- Would graphs help? Would pictures help?

1. Would you actually want to use a computer system if it were available?

- Why/ why not
- At what stage of diagnosis would you find this useful?

**Wrapping up**

1. Is there anything else that you would like to tell me or ask me?

## Asthma CDSS Qualitative Study: Patient Topic Guide

**Experiences of diagnosis**

1. Can you describe your [child’s] route to diagnosis

- Who diagnosed you/them?
- How confident did you feel in their diagnosis?

1. Did you have an idea that you/your child might have asthma before you attended the GP?

- Had you googled symptoms?
- What did you know/think about asthma before the diagnosis?
- Do you know anyone with asthma?
- Did you think it could potentially be something else?
- **IF YES** Do you still think that it could be something else?
- Did you speak to your GP about the possibility of it being something else?

**Tests**

1. During an appointment about your asthma have you ever looked at the computer alongside your GP?

- **IF YES** can you describe the situation in which you were able to view it?
- Who suggested that you look at the computer together?
- What were you able to see?
- Were you able to use it to your advantage to understand more about what your GP was telling you? Why/why not?
- **IF NO** why have you never looked at it?

1. Can you think of a conversation with your GP in which you might have felt this was useful?
2. Did you feel part of the diagnosis process? Why?

- What did your GP do/say that made you feel included?
- What did your GP do/say that made you feel excluded?

1. What do you think GPs could do differently to improve the patient experience of diagnosis in future?
2. Can you think of an example that would have been improved if your GP had managed it differently?

**Uncertainty**

1. Having read about the CDSS, would knowing that your GP is using a diagnostic aid help you to feel more confident in the diagnosis?
2. Why/why not?
3. Would seeing the computer alongside your GP make you feel more/less confident in your diagnosis?
4. Why/why not?
5. What would it be useful for the CDSS to be able to show patients?

- Would graphs help? Would pictures help?

1. Would you actually want to use a CDSS if it were available?

- Why/ why not
- At what stage of diagnosis would you find this useful?

**Wrapping up**

1. Is there anything else that you would like to tell me or ask me?

## Supplementary Table: COREQ (COnsolidated criteria for REporting Qualitative research) Checklist

| **Topic** | **Item No.** | **Guide Questions/Description** | **Page No.** |
| --- | --- | --- | --- |
| **Domain 1: Research team and reﬂexivity** | | | |
| *Personal characteristics* | | | |
| Interviewer/facilitator | 1 | Which author/s conducted the interview or focus group? | 7 |
| Credentials | 2 | What were the researcher’s credentials? E.g. PhD, MD | 7 |
| Occupation | 3 | What was their occupation at the time of the study? | 7 |
| Gender | 4 | Was the researcher male or female? | 7 |
| Experience and training | 5 | What experience or training did the researcher have? | 7 |
| *Relationship with participants* | | | |
| Relationship established | 6 | Was a relationship established prior to study commencement? | No |
| Participant knowledge of  the interviewer | 7 | What did the participants know about the researcher? e.g. personal  goals, reasons for doing the research | None |
| Interviewer characteristics | 8 | What characteristics were reported about the interviewer/facilitator?  e.g. Bias, assumptions, reasons and interests in the research topic | 21 |
| **Domain 2: Study design** | | | |
| *Theoretical framework* | | | |
| Methodological orientation and Theory | 9 | What methodological orientation was stated to underpin the study? | 6 |
| *Participant selection* | | | |
| Sampling | 10 | How were participants selected? | 6 |
| Method of approach | 11 | How were participants approached? e.g. telephone, mail, Email | 6,7 |
| Sample size | 12 | How many participants were in the study? | 8 |
| Non-participation | 13 | How many people refused to participate or dropped out? Reasons? | 8,9 |
| *Setting* | | | |
| Setting of data collection | 14 | Where was the data collected? e.g. home, clinic, workplace | 7 |
| Presence of non-  participants | 15 | Was anyone else present besides the participants and researchers? | No |
| Description of sample | 16 | What are the important characteristics of the sample? e.g. demographic data, date | 6,7,8,9 |
| *Data collection* | | | |
| Interview guide | 17 | Were questions, prompts, guides provided by the authors? Was it pilot tested? | 7 |
| Repeat interviews | 18 | Were repeat interviews carried out? If yes, how many? | 7 |
| Audio/visual recording | 19 | Did the research use audio or visual recording to collect the data? | 7 |
| Field notes | 20 | Were ﬁeld notes made during and/or after the interview or focus group? | no |
| Duration | 21 | What was the duration of the interviews or focus group? | 7 |
| Data saturation | 22 | Was data saturation discussed? | 21 |
| Transcripts returned | 23 | Were transcripts returned to participants for comment and/or correction? | 7 |
| **Domain 3: analysis and ﬁndings** | | | |
| *Data analysis* | | | |
| Number of data coders | 24 | How many data coders coded the data? | 8 |
| Description of the coding  tree | 25 | Did authors provide a description of the coding tree? | 8 |
| Derivation of themes | 26 | Were themes identiﬁed in advance or derived from the data? | 8 |
| Software | 27 | What software, if applicable, was used to manage the data? | 8 |
| Participant checking | 28 | Did participants provide feedback on the ﬁndings? | No |
| Quotations presented | 29 | Were participant quotations presented to illustrate the themes/ﬁndings? Was each quotation identiﬁed? | 10-18 |
| Data and ﬁndings consistent | 30 | Was there consistency between the data presented and the ﬁndings? | 18-20 |
| Clarity of major themes | 31 | Were major themes clearly presented in the ﬁndings? | 10 |
| Clarity of minor themes | 32 | Is there a description of diverse cases or discussion of minor themes? | 21 |
| Developed from: Tong A, Sainsbury P, Craig J. Consolidated criteria for reporting qualitative research (COREQ): a 32-item checklist for interviews and focus groups. *International Journal for Quality in Health Care*. 2007. Volume 19, Number 6: pp. 349 – 357 | | | |
